# 3,4-Di­methyl­phenyl quinoline-2-carboxyl­ate

**DOI:** 10.1107/S1600536813032157

**Published:** 2013-11-30

**Authors:** E. Fazal, Manpreet Kaur, B. S. Sudha, S. Nagarajan, Jerry P. Jasinski

**Affiliations:** a Department of Chemistry, Yuvaraja’s College, Mysore 570 005, India; b Department of Studies in Chemistry, University of Mysore, Manasagangotri, Mysore 570 006, India; c P.P.S.F.T. Department, Central Food Technplogy Research institute, Mysore 570 005, India; d Department of Chemistry, Keene State College, 229 Main Street, Keene, NH 03435-2001, USA

## Abstract

In the title compound, C_18_H_15_NO_2_, the dihedral angle between the mean planes of the quinoline ring system and the phenyl ring is 48.1 (5)°. The mean plane of the carboxyl­ate group is twisted from the mean planes of the latter by 19.8 (8) and 64.9 (5)°, respectively. The crystal packing features weak C—H⋯O inter­actions, which form chains along [010].

## Related literature

For heterocycles in natural products, see: Morimoto *et al.* (1991 ▶); Michael (1997 ▶). For heterocycles in fragrances and dyes, see: Padwa *et al.* (1999 ▶). For heterocycles in biologically active compounds, see: Markees *et al.* (1970 ▶); Campbell *et al.* (1988 ▶). For the use of quinoline alkaloids as drugs for the treatment of malaria, see: Robert & Meunier (1998 ▶). For quinoline as a privileged scaffold in cancer drug discovery, see: Solomon & Lee (2011 ▶). For related structures, see: Fazal *et al.* (2012 ▶); Butcher *et al.* (2007 ▶); Jing & Qin (2008 ▶); Jasinski *et al.* (2010 ▶). For standard bond lengths, see: Allen *et al.* (1987 ▶).
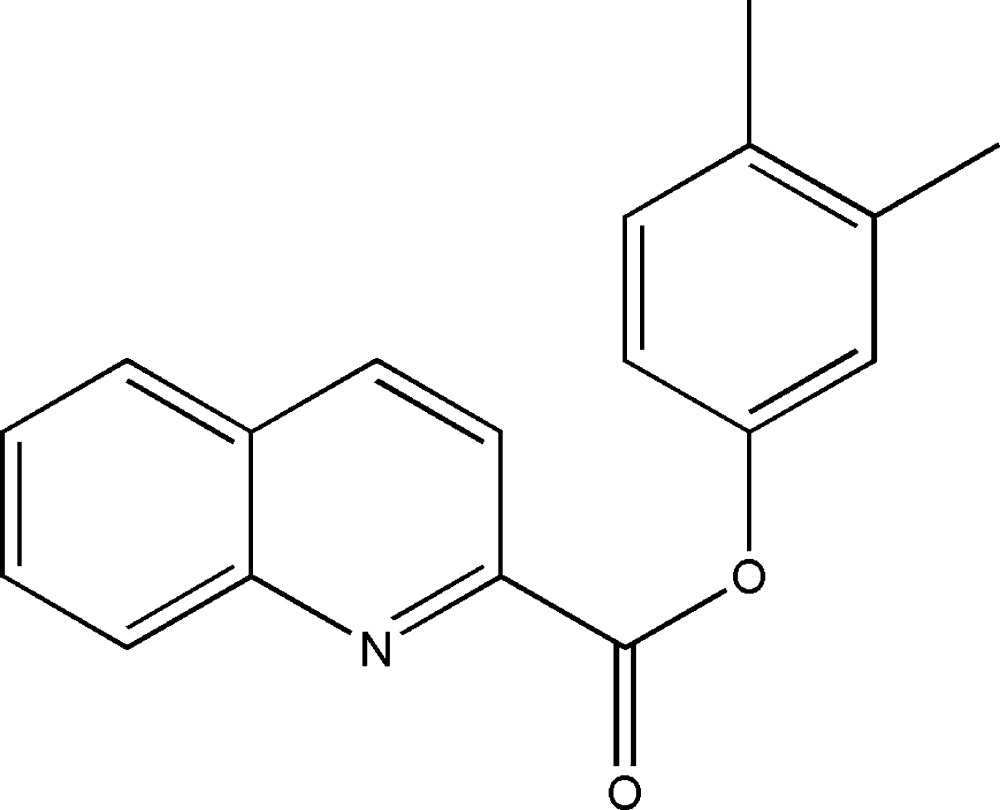


## Experimental

### 

#### Crystal data

C_18_H_15_NO_2_
*M* *_r_* = 277.32
Monoclinic, 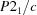

*a* = 6.19172 (17) Å
*b* = 15.4196 (4) Å
*c* = 14.6585 (4) Å
β = 90.761 (3)°
*V* = 1399.38 (7) Å^3^
*Z* = 4
Cu *K*α radiation
μ = 0.69 mm^−1^
*T* = 173 K
0.44 × 0.22 × 0.16 mm

#### Data collection

Agilent Xcalibur (Eos, Gemini) diffractometer
Absorption correction: multi-scan (*CrysAlis PRO* and *CrysAlis RED*; Agilent, 2012 ▶) *T*_min_ = 0.921, *T*_max_ = 1.000
8355 measured reflections
2740 independent reflections
2387 reflections with *I* > 2σ(*I*)
*R*_int_ = 0.042

#### Refinement

*R*[*F* ^2^ > 2σ(*F* ^2^)] = 0.042
*wR*(*F* ^2^) = 0.121
*S* = 1.05
2740 reflections
193 parameters
H-atom parameters constrained
Δρ_max_ = 0.28 e Å^−3^
Δρ_min_ = −0.24 e Å^−3^

### 

Data collection: *CrysAlis PRO* (Agilent, 2012 ▶); cell refinement: *CrysAlis PRO*; data reduction: *CrysAlis RED* (Agilent, 2012 ▶); program(s) used to solve structure: *SUPERFLIP* (Palatinus & Chapuis, 2007 ▶); program(s) used to refine structure: *SHELXL2012* (Sheldrick, 2008 ▶); molecular graphics: *OLEX2* (Dolomanov *et al.*, 2009 ▶); software used to prepare material for publication: *OLEX2*.

## Supplementary Material

Crystal structure: contains datablock(s) I. DOI: 10.1107/S1600536813032157/qm2101sup1.cif

Structure factors: contains datablock(s) I. DOI: 10.1107/S1600536813032157/qm2101Isup2.hkl

Supplementary material file. DOI: 10.1107/S1600536813032157/qm2101Isup3.cml

Additional supplementary materials:  crystallographic information; 3D view; checkCIF report

## Figures and Tables

**Table 1:** Hydrogen-bond geometry (Å, °)

*D*—H⋯*A*	*D*—H	H⋯*A*	*D*⋯*A*	*D*—H⋯*A*
C8—H8⋯O1^i^	0.93	2.48	3.2735 (16)	144

Symmetry code: (i) 
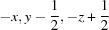
.

**Fig. 1.**
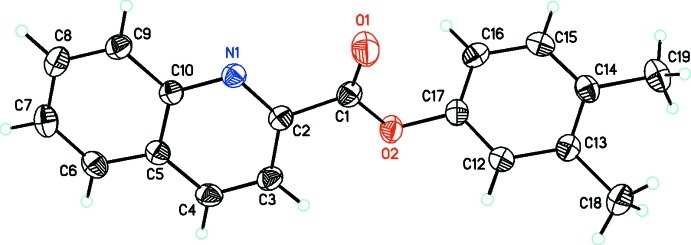
ORTEP drawing of (I) (C18H15NO2) showing the labeling scheme with 50% probability displacement ellipsoids.

**Fig. 2.**
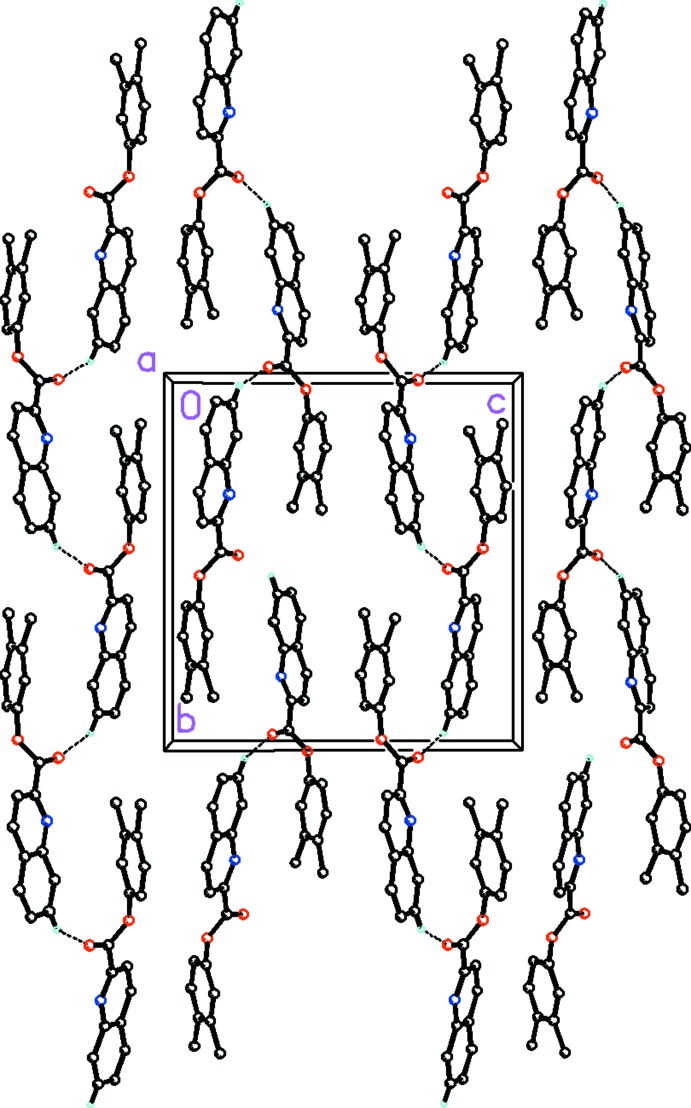
Molecular packing for (I) viewed along the a axis. Dashed lines indicate weak C8—H8···O1 intermolecular interactions making chains along [0 1 0] and influence the crystal packing. The remaining H atoms have been removed for clarity.

## supplementary crystallographic information

### 1. Comment

Quinoline-2 carboxylic acid derivatives are a class of important materials as anti-tuberculosis agents, as fluorescent reagents, hydrophobic field-detection reagents, visualisation reagents, fluorescent labelled peptide probes and as antihyperglycemics. Quinoline derivatives represent a major class of heterocycles and are found in natural products(Morimoto *et al.*, 1991; Michael, 1997), numerous commercial products, including fragrances, dyes(Padwa *et al.*, 1999)and biologically active compounds (Markees *et al.*, 1970; Campbell *et al.*, 1988). Quinoline alkaloids such as quinine,chloroquin, mefloquine and amodiaquine are used as efficient drugs for the treatment of malaria (Robert & Meunier,1998). Quinoline has been used as a privileged scaffold in cancer drug discovery (Solomon & Lee, 2011). The crystal structures of 4-methylphenyl quinoline-2-carboxylate (Fazal *et al.*,2012), 1-(quinolin-2-yl)ethanone(Butcher *et al.*, 2007) and methyl quinoline-2-carboxylate (Jing & Qin, 2008) and the synthesis, crystal structures and theoretical studies of four Schiff bases derived from 4-hydrazinyl-8-(trifluoromethyl) quinoline (Jasinski *et al.*, 2010) have been reported. In view of the importance of quinolines, this paper reports the crystal structure of the title compound, (I), C_18_H_15_NO_2_.

In the title compound, C_18_H_15_NO_2_, the dihedral angle between the mean planes of the quinoline ring and the phenyl ring is 48.1 (5)° (Fig. 1). The mean plane of the carboxylate group is twisted from the mean planes of the quinoline ring and phenyl ring by 19.8 (8)° and 64.9 (5)°, respectively. The crystal packing is influenced by weak C8—H8···O1 intermolecular interactions making chains along [0 1 0](Fig. 2). No classical hydrogen bonds were observed.

### 2. Experimental

The title compound was prepared by the following procedure: To a mixture of 1.73 g (10 mmole) of quinaldic acid and 1.56 g (10 mmole) of 3,4-dimethylphenol in a round-bottomed flask fitted with a reflex condenser with a drying tube is added 0.15 g (10 mmole) of phosphorous oxychloride. The mixture is heated with occasional swirling, and temperature is maintained at 348-353 K. At the end of eight hours the reaction mixture is poured in to a solution of 2 g of sodium bicarbonate in 25 mL of water. The precipitated ester is collected on a filter and washed with water. The yield of crude, air dried 3,4-dimethyl phenyl quinoline-2-carboxylate is 1.47 to 1.90 g (50-65%). X-ray quality crystal was obtained by recrystallization from absolute ethanol.(M.P.:397 K)

### 3. Refinement

All of the H atoms were placed in their calculated positions and then refined using the riding model with Atom—H lengths of 0.93Å (CH) or 0.96Å (CH_3_). Isotropic displacement parameters for these atoms were set to 1.2 (CH) or 1.5 (CH_3_) times *U*_eq_ of the parent atom. Idealised Me refined as rotating group.

### Crystal data

**Table:**

C_18_H_15_NO_2_	*F*(000) = 608
*M**_r_* = 277.32	*D*_x_ = 1.316 Mg m^−^^3^
Monoclinic, *P*2_1_/*c*	Cu *K*α radiation, λ = 1.54184 Å
*a* = 6.19172 (17) Å	Cell parameters from 6294 reflections
*b* = 15.4196 (4) Å	θ = 4.7–72.3°
*c* = 14.6585 (4) Å	µ = 0.69 mm^−^^1^
β = 90.761 (3)°	*T* = 173 K
*V* = 1399.38 (7) Å^3^	Irregular, clear red
*Z* = 4	0.44 × 0.22 × 0.16 mm

### Data collection

**Table:**

Agilent Xcalibur (Eos, Gemini) diffractometer	2740 independent reflections
Radiation source: Enhance (Cu) X-ray Source	2387 reflections with *I* > 2σ(*I*)
Detector resolution: 16.0416 pixels mm^-1^	*R*_int_ = 0.042
ω scans	θ_max_ = 72.3°, θ_min_ = 4.2°
Absorption correction: multi-scan (*CrysAlis PRO* and *CrysAlis RED*; Agilent, 2012)	*h* = −7→6
*T*_min_ = 0.921, *T*_max_ = 1.000	*k* = −18→19
8355 measured reflections	*l* = −14→18

### Refinement

**Table:**

Refinement on *F*^2^	Hydrogen site location: inferred from neighbouring sites
Least-squares matrix: full	H-atom parameters constrained
*R*[*F*^2^ > 2σ(*F*^2^)] = 0.042	*w* = 1/[σ^2^(*F*_o_^2^) + (0.0716*P*)^2^ + 0.1805*P*] where *P* = (*F*_o_^2^ + 2*F*_c_^2^)/3
*wR*(*F*^2^) = 0.121	(Δ/σ)_max_ < 0.001
*S* = 1.05	Δρ_max_ = 0.28 e Å^−^^3^
2740 reflections	Δρ_min_ = −0.24 e Å^−^^3^
193 parameters	Extinction correction: *SHELXL2012* (Sheldrick, 2008), Fc^*^=kFc[1+0.001xFc^2^λ^3^/sin(2θ)]^-1/4^
0 restraints	Extinction coefficient: 0.0048 (6)
Primary atom site location: structure-invariant direct methods	

### Special details

**Table:**

Experimental. ^1^H NMR(500 MHz,DMSO) δ 8.66 (1H,d, J= 8.5Hz), 8.26(1H,d, J= 8.5Hz),8.24(1H,d, J= 8.5 Hz), 8.15(1H,d, J= 8.03 Hz),7.93(1H,dt, J1= 8.2Hz, J2=6.46, J3=1.08Hz), 7.8(1H,t, J= 7.5Hz), 7.25(1H,d, J= 8.2Hz), 7.14(1H,d, J= 2.15Hz), 7.06(1H,dd, J1= 8.03Hz,J2=2.35),2.28(3H,s),2.25(3H,s).
Geometry. All esds (except the esd in the dihedral angle between two l.s. planes) are estimated using the full covariance matrix. The cell esds are taken into account individually in the estimation of esds in distances, angles and torsion angles; correlations between esds in cell parameters are only used when they are defined by crystal symmetry. An approximate (isotropic) treatment of cell esds is used for estimating esds involving l.s. planes.

### Fractional atomic coordinates and isotropic or equivalent isotropic displacement parameters (Å^2^)

**Table:**

	*x*	*y*	*z*	*U*_iso_*/*U*_eq_	
O1	0.03672 (17)	0.48206 (6)	0.20779 (8)	0.0457 (3)	
O2	0.26816 (14)	0.53658 (5)	0.10569 (6)	0.0302 (2)	
N1	0.23607 (16)	0.32280 (7)	0.18546 (7)	0.0247 (2)	
C1	0.19203 (19)	0.47407 (8)	0.16176 (8)	0.0269 (3)	
C2	0.33036 (19)	0.39405 (8)	0.15562 (8)	0.0247 (3)	
C3	0.54041 (19)	0.39753 (8)	0.11950 (8)	0.0286 (3)	
H3	0.5996	0.4500	0.1010	0.034*	
C4	0.65466 (19)	0.32226 (8)	0.11227 (8)	0.0287 (3)	
H4	0.7945	0.3229	0.0899	0.034*	
C5	0.55877 (19)	0.24325 (8)	0.13906 (8)	0.0258 (3)	
C6	0.6605 (2)	0.16151 (9)	0.12935 (9)	0.0315 (3)	
H6	0.7976	0.1583	0.1044	0.038*	
C7	0.5586 (2)	0.08740 (9)	0.15634 (9)	0.0358 (3)	
H7	0.6258	0.0340	0.1487	0.043*	
C8	0.3518 (2)	0.09127 (8)	0.19569 (9)	0.0339 (3)	
H8	0.2852	0.0404	0.2146	0.041*	
C9	0.2485 (2)	0.16894 (8)	0.20625 (9)	0.0284 (3)	
H9	0.1129	0.1707	0.2328	0.034*	
C10	0.34768 (19)	0.24685 (8)	0.17682 (8)	0.0240 (3)	
C12	0.26169 (19)	0.69008 (8)	0.12548 (8)	0.0254 (3)	
H12	0.3995	0.6863	0.1512	0.031*	
C13	0.1647 (2)	0.77085 (8)	0.11323 (8)	0.0263 (3)	
C14	−0.0431 (2)	0.77561 (8)	0.07407 (8)	0.0280 (3)	
C15	−0.1486 (2)	0.69903 (9)	0.04954 (8)	0.0296 (3)	
H15	−0.2870	0.7020	0.0243	0.036*	
C16	−0.0521 (2)	0.61829 (8)	0.06192 (8)	0.0287 (3)	
H16	−0.1240	0.5676	0.0453	0.034*	
C17	0.1535 (2)	0.61561 (8)	0.09951 (8)	0.0257 (3)	
C18	0.2807 (2)	0.85201 (9)	0.14296 (10)	0.0362 (3)	
H18A	0.4197	0.8370	0.1681	0.054*	
H18B	0.1974	0.8813	0.1884	0.054*	
H18C	0.2989	0.8895	0.0913	0.054*	
C19	−0.1517 (2)	0.86210 (9)	0.05940 (10)	0.0402 (3)	
H19A	−0.1732	0.8897	0.1173	0.060*	
H19B	−0.2888	0.8535	0.0294	0.060*	
H19C	−0.0622	0.8981	0.0222	0.060*	

### Atomic displacement parameters (Å^2^)

**Table:**

	*U*^11^	*U*^22^	*U*^33^	*U*^12^	*U*^13^	*U*^23^
O1	0.0474 (6)	0.0355 (5)	0.0550 (7)	0.0099 (4)	0.0268 (5)	0.0120 (4)
O2	0.0339 (5)	0.0233 (5)	0.0335 (5)	0.0028 (3)	0.0083 (4)	0.0041 (3)
N1	0.0242 (5)	0.0260 (5)	0.0240 (5)	−0.0007 (4)	0.0019 (4)	0.0017 (4)
C1	0.0291 (6)	0.0257 (6)	0.0258 (6)	−0.0021 (5)	0.0025 (5)	0.0001 (5)
C2	0.0269 (6)	0.0256 (6)	0.0215 (6)	−0.0014 (4)	0.0000 (4)	0.0012 (4)
C3	0.0282 (6)	0.0287 (6)	0.0290 (6)	−0.0052 (5)	0.0021 (5)	0.0046 (5)
C4	0.0223 (6)	0.0356 (7)	0.0282 (6)	−0.0012 (5)	0.0040 (5)	0.0033 (5)
C5	0.0259 (6)	0.0295 (6)	0.0221 (6)	0.0010 (5)	−0.0008 (4)	0.0010 (4)
C6	0.0290 (6)	0.0362 (7)	0.0293 (6)	0.0059 (5)	0.0024 (5)	0.0002 (5)
C7	0.0438 (8)	0.0273 (7)	0.0362 (7)	0.0085 (5)	−0.0010 (6)	−0.0010 (5)
C8	0.0420 (7)	0.0246 (6)	0.0352 (7)	−0.0038 (5)	−0.0012 (6)	0.0027 (5)
C9	0.0276 (6)	0.0290 (6)	0.0288 (6)	−0.0035 (5)	0.0019 (5)	0.0021 (5)
C10	0.0250 (6)	0.0256 (6)	0.0214 (6)	−0.0004 (4)	−0.0007 (4)	0.0010 (4)
C12	0.0253 (6)	0.0283 (6)	0.0228 (6)	0.0005 (5)	0.0009 (5)	0.0003 (4)
C13	0.0314 (6)	0.0255 (6)	0.0221 (6)	−0.0004 (5)	0.0041 (5)	−0.0012 (4)
C14	0.0306 (6)	0.0304 (6)	0.0230 (6)	0.0058 (5)	0.0047 (5)	0.0006 (5)
C15	0.0249 (6)	0.0390 (7)	0.0250 (6)	0.0009 (5)	0.0004 (5)	0.0012 (5)
C16	0.0316 (6)	0.0290 (6)	0.0255 (6)	−0.0064 (5)	0.0026 (5)	−0.0012 (5)
C17	0.0304 (6)	0.0237 (6)	0.0231 (6)	0.0022 (4)	0.0058 (5)	0.0016 (4)
C18	0.0433 (8)	0.0278 (7)	0.0374 (7)	−0.0023 (5)	−0.0016 (6)	−0.0038 (5)
C19	0.0420 (8)	0.0372 (8)	0.0415 (8)	0.0129 (6)	0.0024 (6)	0.0027 (6)

### Geometric parameters (Å, º)

**Table:**

O1—C1	1.1885 (15)	C9—H9	0.9300
O2—C1	1.3554 (14)	C9—C10	1.4191 (16)
O2—C17	1.4127 (14)	C12—H12	0.9300
N1—C2	1.3214 (15)	C12—C13	1.3933 (17)
N1—C10	1.3665 (15)	C12—C17	1.3806 (17)
C1—C2	1.5053 (16)	C13—C14	1.4039 (18)
C2—C3	1.4118 (17)	C13—C18	1.5045 (17)
C3—H3	0.9300	C14—C15	1.3943 (18)
C3—C4	1.3640 (17)	C14—C19	1.5077 (17)
C4—H4	0.9300	C15—H15	0.9300
C4—C5	1.4132 (17)	C15—C16	1.3918 (18)
C5—C6	1.4170 (17)	C16—H16	0.9300
C5—C10	1.4272 (17)	C16—C17	1.3809 (18)
C6—H6	0.9300	C18—H18A	0.9600
C6—C7	1.3666 (19)	C18—H18B	0.9600
C7—H7	0.9300	C18—H18C	0.9600
C7—C8	1.4122 (19)	C19—H19A	0.9600
C8—H8	0.9300	C19—H19B	0.9600
C8—C9	1.3676 (18)	C19—H19C	0.9600
			
C1—O2—C17	118.28 (9)	C9—C10—C5	119.16 (11)
C2—N1—C10	117.11 (10)	C13—C12—H12	120.0
O1—C1—O2	124.13 (11)	C17—C12—H12	120.0
O1—C1—C2	125.75 (11)	C17—C12—C13	120.08 (11)
O2—C1—C2	110.12 (10)	C12—C13—C14	119.38 (11)
N1—C2—C1	114.05 (10)	C12—C13—C18	120.20 (11)
N1—C2—C3	124.68 (11)	C14—C13—C18	120.42 (11)
C3—C2—C1	121.26 (10)	C13—C14—C19	120.60 (12)
C2—C3—H3	120.7	C15—C14—C13	119.01 (11)
C4—C3—C2	118.56 (11)	C15—C14—C19	120.39 (12)
C4—C3—H3	120.7	C14—C15—H15	119.2
C3—C4—H4	120.3	C16—C15—C14	121.67 (11)
C3—C4—C5	119.46 (11)	C16—C15—H15	119.2
C5—C4—H4	120.3	C15—C16—H16	121.0
C4—C5—C6	123.37 (11)	C17—C16—C15	118.08 (11)
C4—C5—C10	117.68 (11)	C17—C16—H16	121.0
C6—C5—C10	118.95 (11)	C12—C17—O2	117.27 (11)
C5—C6—H6	119.8	C12—C17—C16	121.77 (11)
C7—C6—C5	120.48 (12)	C16—C17—O2	120.75 (11)
C7—C6—H6	119.8	C13—C18—H18A	109.5
C6—C7—H7	119.7	C13—C18—H18B	109.5
C6—C7—C8	120.50 (12)	C13—C18—H18C	109.5
C8—C7—H7	119.7	H18A—C18—H18B	109.5
C7—C8—H8	119.6	H18A—C18—H18C	109.5
C9—C8—C7	120.74 (12)	H18B—C18—H18C	109.5
C9—C8—H8	119.6	C14—C19—H19A	109.5
C8—C9—H9	119.9	C14—C19—H19B	109.5
C8—C9—C10	120.13 (11)	C14—C19—H19C	109.5
C10—C9—H9	119.9	H19A—C19—H19B	109.5
N1—C10—C5	122.42 (10)	H19A—C19—H19C	109.5
N1—C10—C9	118.42 (11)	H19B—C19—H19C	109.5
			
O1—C1—C2—N1	18.18 (18)	C8—C9—C10—N1	177.64 (11)
O1—C1—C2—C3	−162.66 (13)	C8—C9—C10—C5	−2.07 (18)
O2—C1—C2—N1	−160.96 (10)	C10—N1—C2—C1	176.20 (10)
O2—C1—C2—C3	18.20 (16)	C10—N1—C2—C3	−2.92 (18)
N1—C2—C3—C4	1.69 (19)	C10—C5—C6—C7	−0.47 (19)
C1—O2—C17—C12	118.93 (12)	C12—C13—C14—C15	0.89 (18)
C1—O2—C17—C16	−66.23 (14)	C12—C13—C14—C19	−179.61 (11)
C1—C2—C3—C4	−177.37 (11)	C13—C12—C17—O2	174.15 (10)
C2—N1—C10—C5	1.18 (17)	C13—C12—C17—C16	−0.63 (18)
C2—N1—C10—C9	−178.52 (11)	C13—C14—C15—C16	−0.78 (18)
C2—C3—C4—C5	1.36 (18)	C14—C15—C16—C17	−0.04 (18)
C3—C4—C5—C6	176.44 (12)	C15—C16—C17—O2	−173.85 (10)
C3—C4—C5—C10	−2.87 (18)	C15—C16—C17—C12	0.75 (18)
C4—C5—C6—C7	−179.76 (12)	C17—O2—C1—O1	−1.63 (18)
C4—C5—C10—N1	1.64 (18)	C17—O2—C1—C2	177.53 (10)
C4—C5—C10—C9	−178.66 (11)	C17—C12—C13—C14	−0.21 (18)
C5—C6—C7—C8	−1.0 (2)	C17—C12—C13—C18	178.94 (11)
C6—C5—C10—N1	−177.69 (11)	C18—C13—C14—C15	−178.26 (11)
C6—C5—C10—C9	2.00 (18)	C18—C13—C14—C19	1.24 (18)
C6—C7—C8—C9	1.0 (2)	C19—C14—C15—C16	179.73 (11)
C7—C8—C9—C10	0.58 (19)		

### Hydrogen-bond geometry (Å, º)

**Table:**

*D*—H···*A*	*D*—H	H···*A*	*D*···*A*	*D*—H···*A*
C8—H8···O1^i^	0.93	2.48	3.2735 (16)	144

Symmetry code: (i) −*x*, *y*−1/2, −*z*+1/2.
